# Calibration and verification of DEM parameters of wet-sticky feed raw materials

**DOI:** 10.1038/s41598-023-36482-w

**Published:** 2023-06-07

**Authors:** Fei Peng, Limei Zhang, Zhiqiang Li, Jianming Chen

**Affiliations:** 1 School of Artificial Intelligence, Beijing Technology and Business University, Beijing, 100048 China; 2 Key Laboratory of Healthy Freshwater Aquaculture, Zhejiang Institute of Freshwater Fisheries, Huzhou, 313001 China

**Keywords:** Engineering, Mathematics and computing

## Abstract

In order to improve the accuracy of the parameters needed in the discrete element method (DEM) simulation process of wet-sticky feed raw materials, the JKR contact model in DEM was used to calibrate and verify the physical parameters of wet-sticky feed raw materials. Firstly, the parameters that have a significant effect on the angle of repose were screened using a Plackett–Burman design, and the screened parameters were: MM rolling friction coefficient, MM static friction coefficient, and JKR surface energy. Then, the three screened parameters were selected as the influencing factors and the accumulation angle of repose was selected as evaluating indicator; thus, the performance optimization experiments were carried out with the quadratic orthogonal rotation design. Taking the experimentally measured angle of repose value of 54.25°as the target value, the significance parameters were optimized, and the optimal combination was obtained : MM rolling friction factor was 0.21, MM static friction factor was 0.51, and JKR surface energy was 0.65. Finally, the angle of repose and SPP tests were compared under the calibrated parameters. The results showed that the relative error of experimental and simulated tests in angle of repose was 0.57%, and the compression displacement and compression ratio of the experimental and simulated tests in SPP were 1.01% and 0.95%, respectively, which improved the reliability of the simulated results. The research findings provide a reference basis for simulation study and optimal design of related equipment for feed raw materials.

## Introduction

**Figure 1:**
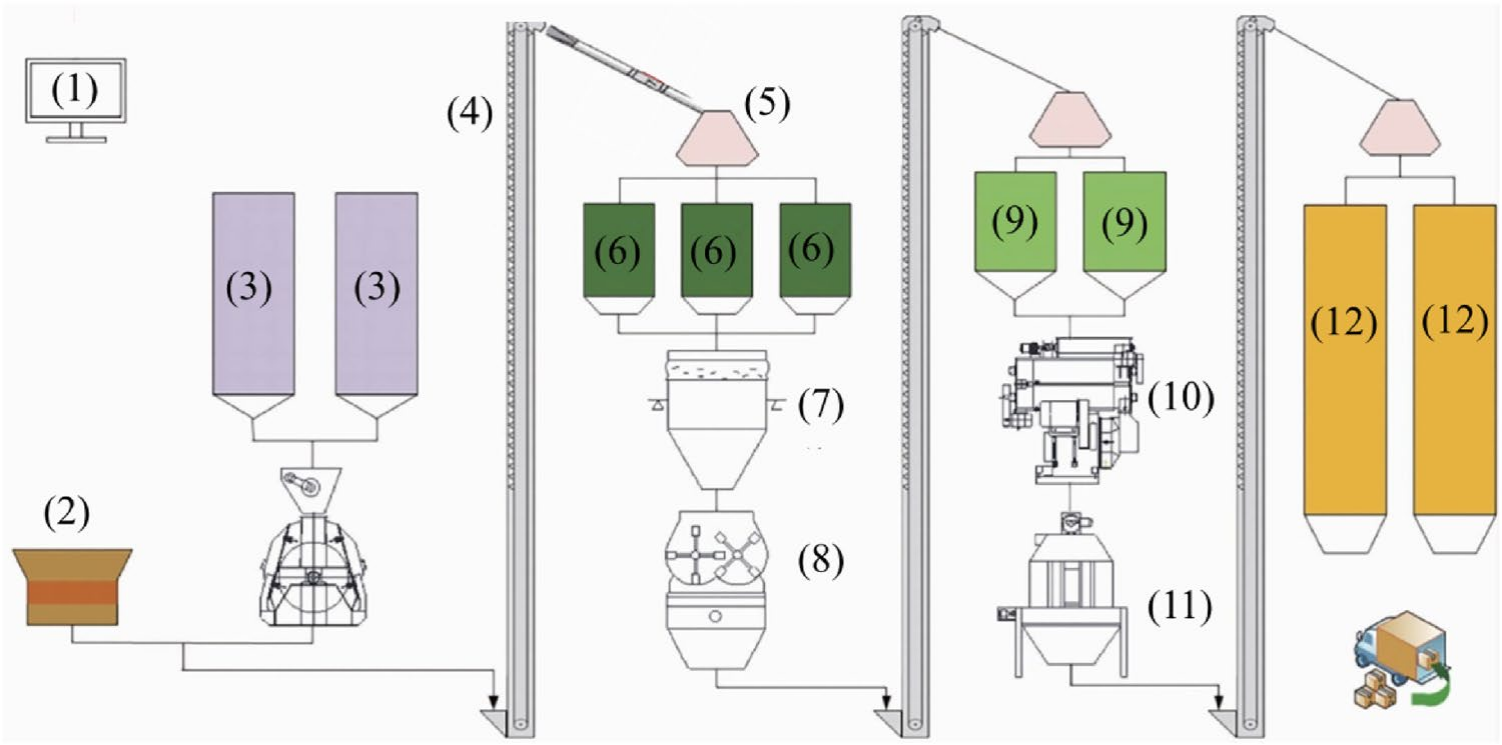
Schematic of feed pelleting process in a bench-scale.

As the main food source of animals, pellet feed is the material basis of livestock and aquaculture. In China, the total feed output reached 162 million tons in 2010, becoming the world's largest feed producer, and it reached 293 million tons in 2021^1^. Feed processing mainly includes receiving, cleaning, crushing, batching, mixing, conditioning, granulating, cooling, packaging and distribution of end products (Fig. 1), which contributed to increase nutrient availability for animals. Among all these processing procedures mentioned, pelleting is the most critical process and has an extraordinarily important influence on feed quality^2^. Pelleting aims to agglomerate ingredients particles by mechanical action, in combination with moisture, pressure and temperature^3,4^. Actually, the essence of feed pelleting is exactly the extrusion and forming process of feed raw materials powder. When pelleting, feed raw materials is in powder form and a wet-sticky state. To study the complicated behavior during the pelleting process is necessary to deeply understand the mechanism of the pelleting process, the performance of pelleting operation and the accurate quantitative analysis. However, the procedures performed by industrials are expensive trial and error. Besides, the pellet mill is in a dynamic rotation state and the pelleting process occurs in a relatively closed room; therefore, it is difficult to obtain data in the process of extrusion and pelleting, which brings an enormous challenge to dig into the mechanical behavior and pelleting mechanism of pellet feed. At present, both isostatic and closed die compressions have been carried out to study particle rearrangement and mechanical behavior^5,6^. Moreover, numerous studies have shown that it is meaningful and feasible to analyze the various mechanical behaviors and state change laws of the feed in the pellet mill by the single pellet press device^7–9^. Therefore, this article implemented a single pellet press (SPP) device for experimental and simulated verification tests.

The simulation technology, providing an interesting insight into the different physical phenomena present, can be considered as an effective and inexpensive solution to analyze pelleting process. The discrete element method, proposed by Professor Cundall from the United States in 1971, is an effective method based on the molecular dynamics principle to analyze discrete particle materials^9^. DEM could provide particles information related to the location, velocity, stress and energy, and so on in powder systems^10–12^. In recent years, DEM has been universally used to study powder dynamics phenomena involving complex physical fields, material mechanical properties with more complex structures and multiphase mixed material medium. More specifically, it has been widely used in compaction or briquetting technologies in similar fields^13,14^, such as coal^15^, rice kernel^16^, tablet^17^, etc. In the DEM calculation, the physical and bonded parameters are principal particle properties, which affect the simulated results. Due to the differences in the physical parameters for various material particles, it is necessary to calibrate the material particles before the simulation to obtain accurate and reliable numerical simulation results. However, feed raw materials are in a wet-sticky state and have very high physical properties (fluidity, adhesion, compressibility and other characteristics), which are different from these materials that are mainly in a natural state. On the other hand, the conventional calibration method of DEM parameters is simply the angle of repose, a relatively indirect index, instead of directly facing the equipment. Thus, when the DEM parameters calibrated with the traditional method are used for simulation, the numerical simulation error is relatively large and the results are unreliable.

In this paper, firstly, JKR contact model was selected considering the cohesion between wet-sticky feed raw materials. The Plackett–Burman design experiment was employed to screen out the significant parameters. Then, based on regression analysis and response surface analysis method, the optimal combination of the influencing factors was obtained. Finally, the calibrated parameters were applied to SPP DEM compression simulation, and the relative error of compression displacement and force between DEM simulated and experimental test was compared and analyzed to verify the feasibility and accuracy of the method.

## Materials and methods

### Analysis of the working process

**Figure 2:**
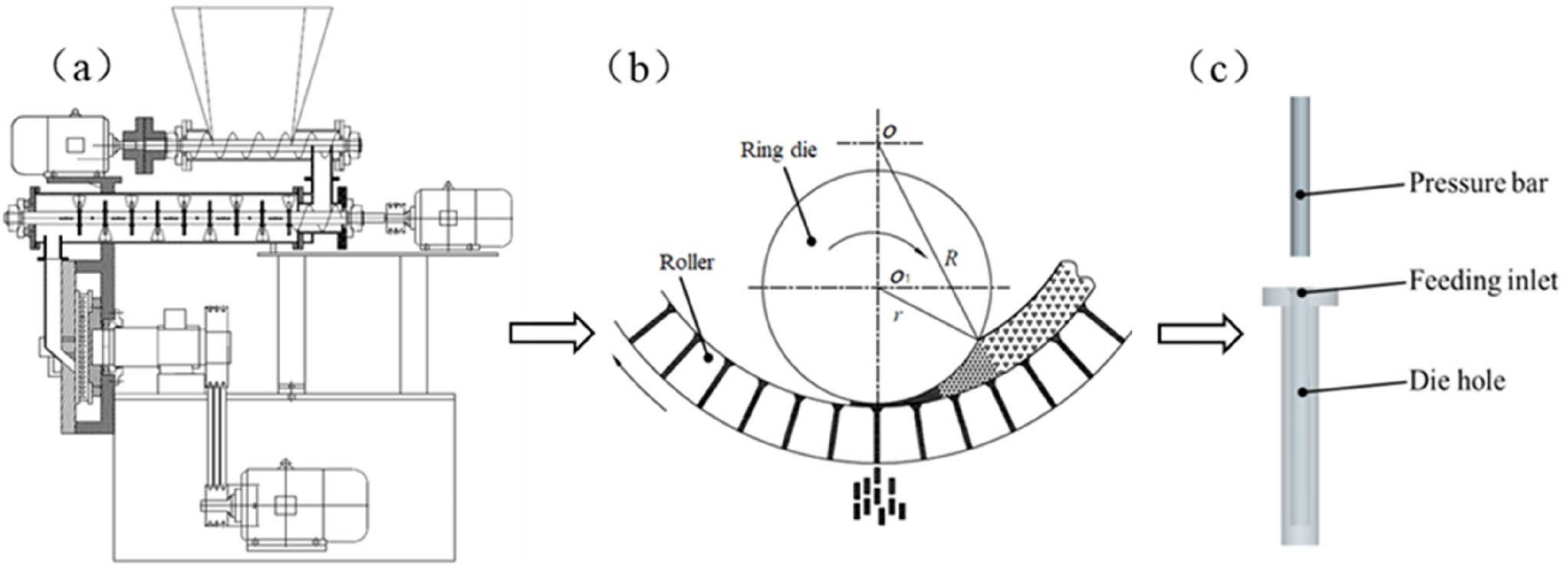
From a pellet mill to its pelleting principle, and then to a single pellet press model: (**a**) a bench-scale pellet mill^18^; (**b**) pelleting principle of the pellet mill; (**c**) a single pellet press model.

The structure of a feed pellet mill was shown in Part 10 of Figs. 1 and 2a. The essence of its work was the change of the interval among powder particles. As exhibited in Fig. 2b, the pelleting principle of the pellet mill was as follows: as the interval reduced, the density and strength of particles increased under the action of compression and extrusion force. When the extrusion force overcame friction force from the inner wall of the ring die, the materials was eventually extruded out of the die hole. To better characterize the extrusion process and simulation, the extrusion process model was simplified to a single pellet press model, as shown in Fig. 2c.

### Contact model selection

**Figure 3:**
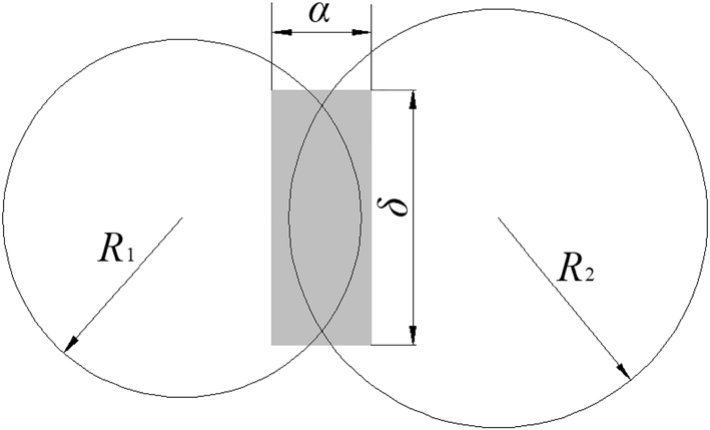
Sketch of JKR contact model.

DEM contact model should be selected based on the corresponding material properties of the simulation object. As the main component of feed raw materials, starch reacted with saturated water vapor during the conditioning process, which caused cohesive force and created appropriate conditions for agglomeration and pelletizing. The feed material was wet and sticky after conditioning and before pelleting, which was typical viscous particles. Therefore, it was necessary to introduce a viscous bond to characterize the interaction mode among the particles. The Hertz-Mindlin with JKR contact model (Fig. 3), suitable for the cohesive particle, was adopted to simulate the cohesive effect among fine and wet particles. The mathematical model^19^ for the JKR contact model was as follows:

1 $$ F_{JKR} = - 4\sqrt {\pi \gamma E^{*} \alpha^{\frac{3}{2}} } + \frac{{4E^{*} }}{{3R^{*} }}\alpha^{3} $$

2 $$ \delta = \frac{{\alpha^{2} }}{{R^{2} }} - \sqrt {\frac{4\pi \gamma \alpha }{{E^{*} }}} $$

3 $$ \frac{1}{{E^{*} }} = \frac{{1 - U_{1}^{2} }}{{E_{1} }} + \frac{{1 - U_{2}^{2} }}{{E_{2} }} $$

4 $$ \frac{1}{{R^{*} }} = \frac{1}{{R_{1} }} + \frac{1}{{R_{2} }} $$

When surface energy γ = 0, normal elastic force of JKR contact model equated to Hertz-Mindlin normal force, given by^20^

5 $$ F_{JKR} = F_{Hertz} = \frac{4}{3}E^{*} \sqrt {R^{*} \delta^{\frac{3}{2}} } $$

The JKR model could still supply the cohesive force even if the particles were not in direct contact. The maximum gap *δ*_*c*_ and critical contact radius *α*_*c*_ of non-zero cohesive force between particles could be reckoned as follows^21^

6 $$ \delta_{c} = \frac{{\alpha_{c}^{2} }}{{R^{*} }} - \sqrt {4\pi \alpha_{c} /E^{*} } $$

7 $$ \alpha_{c} = \left[ {\frac{{9\pi \gamma R^{*} }}{{2E^{*} }}\left( {\frac{3}{4} - \frac{1}{\sqrt 2 }} \right)} \right]^{\frac{1}{3}} $$

where *F*_JKR_ is normal elastic force (N); *α* is normal overlap between the particles (m); *δ* is tangential overlap between the particles (m); *γ* is surface energy (J/m^2^); E^*^ is equivalent elastic modulus (Pa); R^*^ is equivalent contact radius (m); *E*_1_ and* E*_2_ are elastic modulus of contact particles (Pa); *U*_1_ and* U*_2_ are Poisson's ratio of contact particles; *R*_1_ and* R*_2_ are the radius of contact particles (m).

### Simulation parameter selection

**Figure 4:**
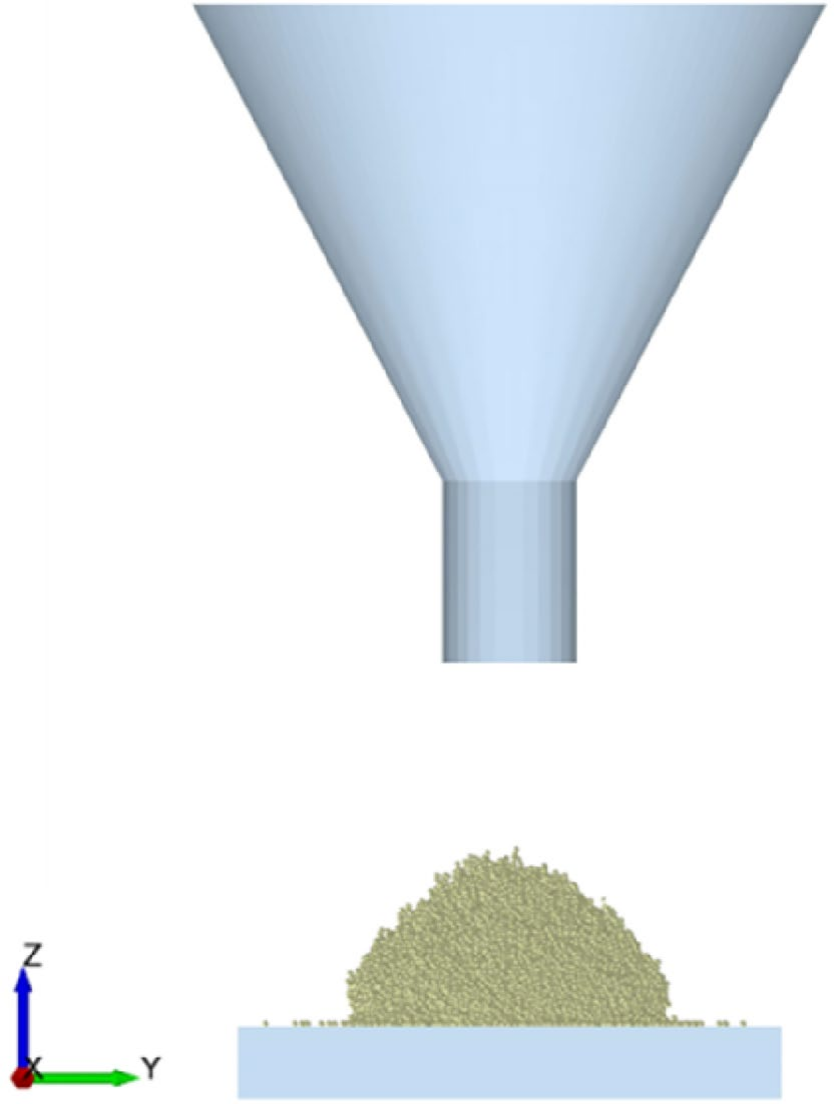
Simulation model of repose of angle for wet-sticky feed raw materials.

Angle of repose was an essential and effective index to calibrate DEM parameters of materials by comparing the virtual simulated and practical experiments. The feed raw material was made by crushing, and its shape was fine particles. Considering the shape and size of feed raw material, the simulation conditions, and time constraints, spherical particles were selected for the simulation. The intrinsic parameters of the wet-sticky feed raw materials required for simulation were set as follows: for the wet-sticky feed raw materials, density was 440 kg/m^3^, Poisson’s ratio was 0.25, particle size was 0.10 mm, shear modulus was 10 × 10^6^ Pa; for stainless steel plate, density was 7850 kg/m^3^, Poisson’s ratio was 0.3, shear modulus was 7 × 10^7^ Pa. The generation method of particles was set to be dynamic, and the particle generation rate was 10,000 per second. In addition, the number of generations was set to 25,000, the simulation time was 8.0 s, the time step was 0.01 s, and the mesh size was 3R. Other physical parameters chosen for simulation were close to reality. The simulation parameters were summarized in Table 1. The DEM parameter calibration process of feed raw materials was as follows: first, a simulation model of repose angle was built, and materials particles dropped freely from the funnel under gravity, forming a stable particle pile, as shown in Fig. 4. Second, the angle of repose value was calculated based on the accumulation of the particle pile accumulation image, which was examined using the protractor function in the tools option. Finally, the angle of repose values from the simulated test and the experimental tests were compared to assess the validity and correctness of the simulation parameters.

**Table 1:** Physical properties of materials and device in DEM model.

Item	Parameters	Values
Particles^22^	Particle size/mm	0.10
	Shear modulus/Pa	10 × 10^6^
	Poisson’s ratio	0.25
	Density/(kg/m^3^)	440
Angle of repose test device (steel^16^)	Shear modulus/Pa	7 × 10^10^
	Poisson’s ratio	0.3
	Density/(kg/m^3^)	7850
Contact parameters	MM coefficient of restitution	0.05–0.3
	MM rolling friction factor	0.1–0.5
	MM static friction factor	0.4–0.8
	MS coefficient of restitution	0.05–0.3
	MS rolling friction factor	0.1–0.4
	MS static friction factor	0.3–0.7
	JKR surface energy/(J/m^2^)	0.1–0.8

Notes: MM means between feed raw materials and materials; MS means between feed raw materials and stainless steel.

#### Plackett–Burman simulation experiment

Based on the relationship between the target response and each factor, Plackett–Burman design was used to determine the significance of the factor by comparing the difference between the two levels of each factor^23,24^. Angle of repose was selected as the target parameter to screen the significance of the simulated contact parameters. The low level referred to the original level, and the high level was set to twice the low level. The test parameters were presented in Table 2.

**Table 2:** Parameters of Plackett–Burman test.

Symbol	Parameters	Low level (−1)	High level (−1)
A	MM coefficient of restitution	0.05	0.1
B	MM rolling friction factor	0.1	0.2
C	MM static friction factor	0.4	0.8
D	MS coefficient of restitution	0.15	0.3
E	MS rolling friction factor	0.1	0.2
F	MS static friction factor	0.3	0.6
G	JKR surface energy/(J/m^2^)	0.1	0.2

## Results and discussion

### Significance analysis of factors in the Plackett–Burman test

The Plackett–Burman test design and results were presented in Table 3. A-G referred to the coded values. The variance analysis of the results was carried out using design expert software, and the significance of each contact parameter was exhibited in Table 4. It showed that the three factors (JKR surface energy, MM static friction factor and MM rolling friction) *P* < 0.01, which indicated the three factors had extremely significant effects on angle of repose; While, the other four factors (MM coefficient of restitution, MS coefficient of restitution, MS rolling friction factor and MS static friction factor) *P* > 0.05, which indicated the four factors had little significant effects on angle of repose. In order to efficiently facilitate the following test, only these three significant factors were considered in the quadratic orthogonal rotation design. The rest of parameters were selected in combination with literature^22,25^. Specifically, MM coefficient of restitution was 0.15, MS coefficient of restitution 0.1, MS rolling friction factor 0.2, and MS static friction factor 0.45.

**Table 3:** Design and results of Plackett–Burman test.

No	A	B	C	D	E	F	G	Angle of repose (°)
1	1	1	1	−1	−1	−1	1	46.9
2	−1	−1	−1	−1	−1	−1	−1	35.2
3	−1	1	−1	1	1	−1	1	42.5
4	1	1	−1	−1	−1	1	−1	38.3
5	1	1	−1	1	1	1	−1	37.5
6	1	−1	−1	−1	1	−1	1	40.4
7	−1	1	1	1	−1	−1	−1	41.8
8	1	−1	1	1	−1	1	1	43.5
9	−1	1	1	−1	1	1	1	46.4
10	1	−1	1	1	1	−1	−1	38.3
11	−1	−1	−1	1	−1	1	1	41.7
12	−1	−1	1	−1	1	1	−1	38.6

**Table 4:** Analysis of parameters of significance.

Parameters	Coefficient	Sum of squares	*F-*value	*P-*value	Significance
A	-0.11	0.14	0.38	0.5694	5
B	1.31	20.54	55.89	0.0017	3
C	1.66	33.00	89.80	0.0007	2
D	-0.042	0.021	0.057	0.8235	7
E	-0.31	1.14	3.10	0.1529	4
F	0.075	0.067	0.18	0.6903	6
G	2.64	83.74	227.87	0.0001	1

### Quadratic regression orthogonal rotating test and regression model

In order to calibrate the JKR contact model of the wet-sticky feed raw materials, virtual simulated tests were conducted using the regression analysis software Design-Expert 8.0.6 and the response surface analysis method. Based on the orthogonal rotation combination test principle, the level coding values of each influencing factor (MM rolling friction factor *X*_1_, MM static friction factor *X*_2_, and JKR surface energy *X*_3_) were selected as the independent variable. The angle of repose, measured by the simulation results, was selected as the evaluation index. The variables and levels were listed in Table 5.

**Table 5:** Factors and codes of quadratic regression orthogonal rotating test.

**Numerical variable**		**Code variable levels**				
	**Symbol**	**-2**	**-1**	**0**	**1**	**2**
MM rolling friction factor	*X*_1_	0.1	0.2	0.3	0.4	0.5
MM static friction factor	*X*_2_	0.3	0.45	0.6	0.75	0.9
JKR surface energy/(J/m^2^)	*X*_3_	0.2	0.35	0.5	0.65	0.8

#### Regression model analysis

The geometric models of different experimental groups were constructed and then imported into DEM for simulation; thus the results were obtained as shown in Table 6.

**Table 6:** Simulated levels and results of quadratic regression orthogonal rotating test.

No	*X*_1_	*X*_2_	*X*_3_	Angle of repose/(°)
1	1	1	1	63.86
2	1	1	-1	44.95
3	1	-1	1	57.42
4	1	-1	-1	38.05
5	-1	1	1	52.39
6	-1	1	-1	41.28
7	-1	-1	1	53.44
8	-1	-1	-1	27.62
9	2	0	0	48.73
10	-2	0	0	35.47
11	0	2	0	68.11
12	0	-2	0	36.90
13	0	0	2	74.55
14	0	0	-2	31.83
15	0	0	0	54.15
16	0	0	0	54.35
17	0	0	0	55.82
18	0	0	0	53.21
19	0	0	0	54.27
20	0	0	0	53.71
21	0	0	0	53.25
22	0	0	0	53.68
23	0	0	0	54.01

The Analysis of variance (ANOVA) for angle of repose was executed by Design-Expert software, as shown in Table 7. The ANOVA showed *P* < 0.01, which indicated the regression was extremely significant. The determination coefficient *R*^2^ was 0.92 and the correction determination coefficient was 0.87, which indicated that the regression equation’s fitting degree was good, and the regression equation could be used to analyze the test results. The significance of the determination coefficient was tested. According to the *P*-value in the regression equation, *X*_1_, *X*_2_ and *X*_3_ had significant effect on the angle of repose. The final quadratic regression model for angle of repose was obtained as Eqs. (8):

8 $$ \begin{aligned} {\text{AOR}} & = {53}.{67} + {3}.{5}0X_{{1}} + {5}.{52}X_{{2}} + {1}0.0{4}X_{{3}} + 0.0{91}X_{{1}} X_{{2}} + 0.{17}X_{{1}} X_{{3}} \\ & \quad - {1}.{9}0X_{{2}} X_{{3}} - {3}.{32}X_{{1}}^{{2}} - 0.{72}X_{{2}}^{{2}} - 0.{55}X_{{3}}^{{2}} \\ \end{aligned} $$

**Table 7:** Analysis of variance (ANOVA) for angle of repose.

Source	SS	DF	MNS	*F-*value	*P-*value
Model	2639.6	9	293.29	23.44	< 0.001**
*X*_1_	196.49	1	196.49	15.70	0.0016**
*X*_2_	488.08	1	488.08	39.00	< 0.001**
*X*_3_	1613.03	1	1613.03	128.90	< 0.001**
*X*_1_* X*_2_	0.07	1	0.07	0.005	0.9429
*X*_1_* X*_3_	0.23	1	0.23	0.018	0.8947
*X*_2_* X*_3_	28.77	1	28.77	22.30	0.1534
*X*_1_^2^	309.77	1	309.77	24.75	0.0003**
*X*_2_^2^	14.51	1	14.51	1.16	0.3011
*X*_3_^2^	8.42	1	8.42	0.67	0.4269
*Residual*	162.68	13	12.51	-	-
Lack of Fit	157.79	5	31.56	51.73	< 0.001
Pure Error	4.88	8	0.61	-	-

**Indicated the significant at P < 0.01. DF referred to degree of freedom; SS referred to sum of squares; DF referred to degree of freedom; MNS referred to mean square.

#### Interaction analysis of regression model

**Figure 5:**
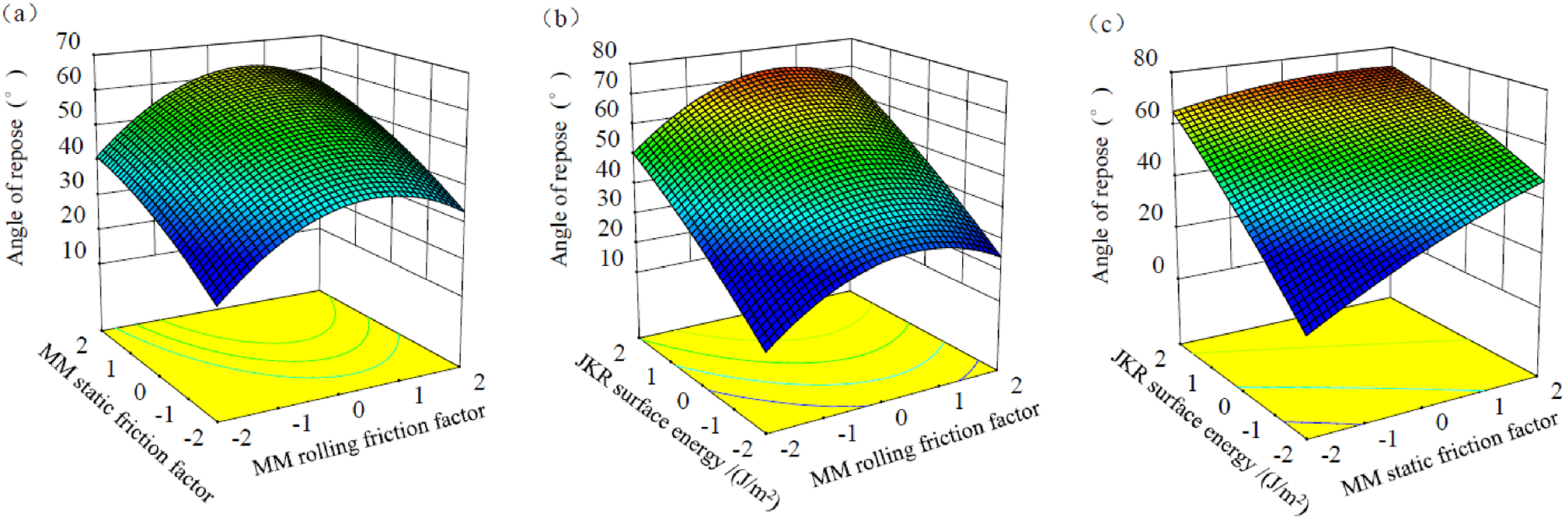
Response surface plots of the angle of repose.

The response surface diagram, drawn by the Design Expert Software, was used to analyze the influence of various factors on angle of repose. By fixing one of the three factors to the zero level successively, the influence law of the other two factors on the angle of repose of raw materials was investigated. As shown in Figs. 5a–c, the interaction effect between the two parameters could be observed intuitively. As depicted in Fig. 5a,b, it showed that angle of repose of feed raw materials increased with the increase of MM rolling friction coefficient. The reason may be that: when the MM rolling friction coefficient value was low, the boundary particles would be pushed out by the central particles during the stacking process, and the boundary diffusion was more obvious; when the MM rolling friction coefficient was high, it was not conducive to the diffusion of boundary particles, and the particles accumulated in the height direction of the particle pile (i.e., z-axis direction). That was the microscopic explanation for that repose of angle increased with the increase of MM rolling friction coefficient. As displayed in Fig. 5a,c., it could be seen that angle of repose of feed raw materials showed an increasing trend with the increase of the MM static friction coefficient between particles. This reason may be due to that when the MM rolling friction coefficient value was high, the friction resistance of the contact part between feed raw materials was greater; thus, the feed raw materials were more difficult to slide and scatter, and the formed accumulation tends to be more stable^26^. Therefore, angle of repose of feed raw materials increased with the increase of the MM rolling friction coefficient. As described in Fig. 5b,c, the repose angle of pellet feed showed an increasing trend with the increase of JKR surface energy. JKR contact model, which was introduced with the concept of surface energy between particles, was suitable for simulating the adhesion between particles of fine and wet materials. When the JKR surface energy value was high, the adhesion reaction between particles was stronger and adhesive forces would enhance.

## Parameter optimization and verification tests

### Angle of repose calibration tests

The target parameter for angle of repose value determined in the practical experiment was 54.25°, and then it was substituted into the Design-Expert 8.0.6 software. The DEM parameters *X*_i_ (i = 1, 2, 3) were further optimized within the range of—2 to 2 by the response surface method. Finally, the optimal calibration parameter combination was obtained as follows: the coded value *X*_1_ was -0.92, *X*_2_ was -0.60, *X*_3_ was 0.97; this is, the true value *x*_1_ was 0.21, *x*_2_ was 0.51, *x*_3_ was 0.65. In this way, the optimal DEM parameters for target repose angle of feed raw materials were obtained. In DEM software, the values of *x*_1_, *x*_2_, and *x*_3_ were set according to the above optimal solution, and the remaining parameters were set to the intermediate level; thus the DEM model of feed ingredients can be obtained. Repeat it 5 times, and the angle of repose values were 54.72°, 53.95°, 53.63°, 53.23°, and 54.18°, respectively. The average angle of repose was 53.94°, and the relative error between the simulated and experimental experiment results was 0.57%; therefore, there was no obvious error between the simulated and experimental tests. The DEM particles were set in the ideal state in simulation, and their surface was smoother than those in the actual test. Therefore, the values of MM sliding friction coefficient and MM rolling friction coefficient calibrated in simulation models were less than that in the experimental test, so as to achieve the high consistency between the simulated and experimental results. Based on the internal relationship and equivalence principle^27^, the angle of repose simulation results in calibrating DEM simulated tests, were basically the same as those in the experimental test.

### SPP tests

**Figure 6:**
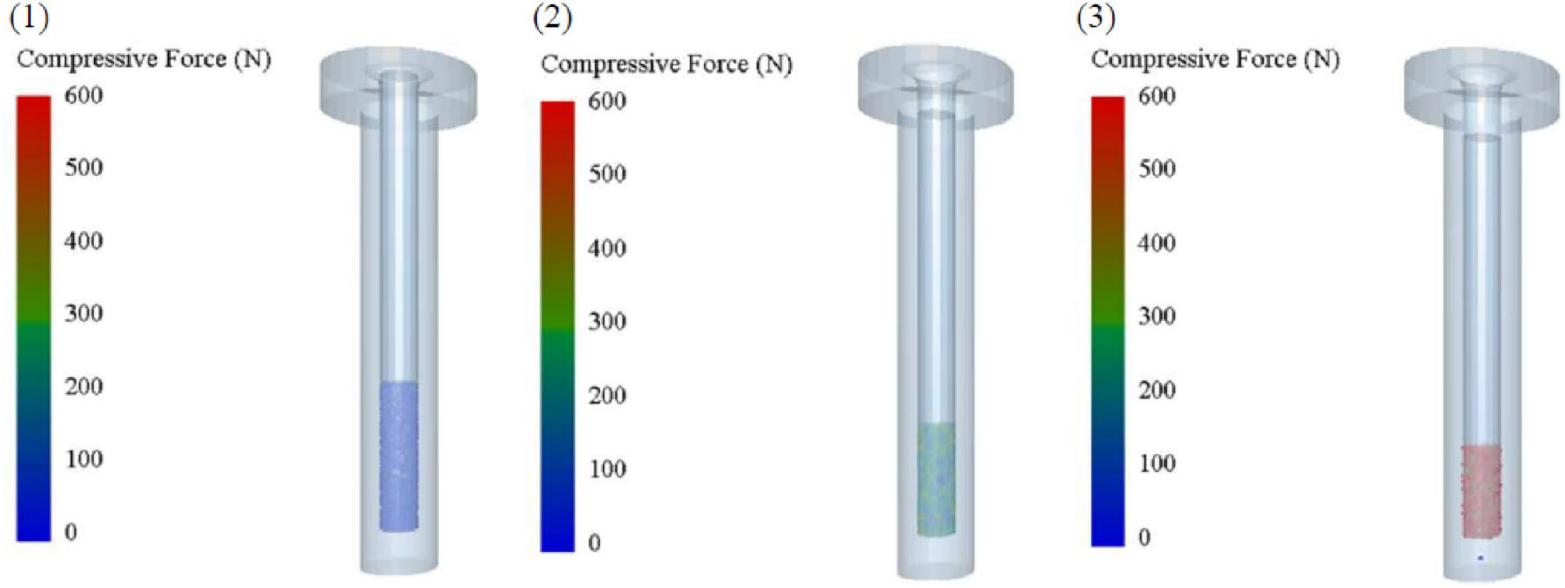
Results of SPP DEM simulation: (1) t = 0 min; (2) t = 0.5 min; (2) t = 1 min.

The single pellet press device was mainly composed of four parts: pressure bar, feeding inlet, die hole, and backstop. The SPP experimental test procedure referred to our previous study^28^. The SPP DEM simulation model was established and simplified, and its simulation process was demonstrated in Fig. 6. In the initial state, the particles were in a loose state and the voids among particles were large. During the compression process, the voids decreased gradually, and then the particles overlapped and interlaced under the action of extrusion force. Later, the gap between particles reduced significantly and the overlap between particles increased sharply, and then some particles were wrapped by the surrounded particles. The phenomenon of the simulated test above was consistent with the actual feed pelleting process, which further verified the rationality of the simulated results. Meanwhile, from the view of extrusion force value and distribution of particles, it could be seen that most of the particles were under the minimum pressure at first, and then the extrusion force of particles increased gradually and the maximum value of extrusion force increased several hundred times the numerical scale as the extrusion process continues. When the extrusion process reached the end, all particles were subjected to a greater extrusion pressure force. The size distribution was uniform, which was consistent with the actual experiment tests.

Compression force was a primary factor used to reflect the pelleting evolution process and affected the quality of pellet feed. Therefore, compression force was selected to verify the availability of the DEM model by comparing the simulated data with the experimental data and calculating the relative error. The simulated data and experimental data generated by extrusion at a compression speed of 20 mm/min, and the compression forces data could be obtained by post-processing module after the test.

**Figure 7:**
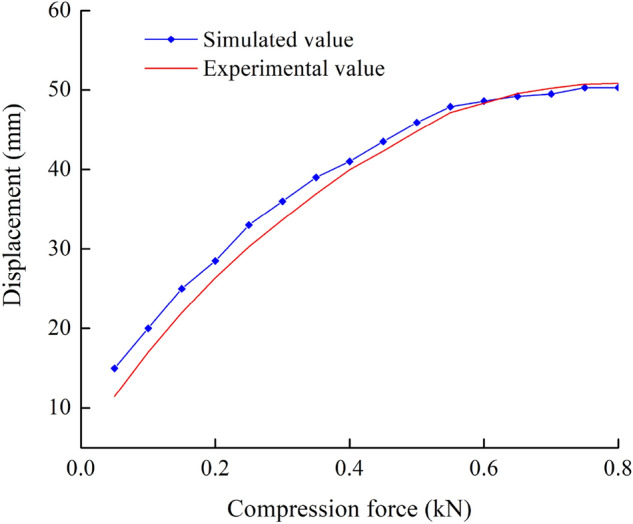
Displacement- Force curve for simulated and experimental values.

As shown in Fig. 7, typical compression forces obtained by both DEM simulated and experimental tests were investigated. From the perspective of the changing trend, the extrusion load curve showed a low value at the initial pelleting stage while increasing rapidly in a nonlinear manner as the powder was compressed and compacted. The simulated and experimental results of the force–displacement curve were consistent. The maximum compression displacement and compression ratio obtained from the simulated test were 50.30 mm and 0.629 respectively, which were close to the experimental test 50.81 mm and 0.635. The relative errors of maximum compression displacement and compression ratio were 1.01% and 0.95%, respectively, which were relatively small. Therefore, the DEM compression tests were in the acceptable range. In addition, the simulated value of the extrusion displacement curve was relatively lower than that in the experimental test. It may be attributed to the fact that the extrusion process of gas in the gap between particles was relatively simplified, making the particles quickly fit and inlay to complete the whole process. As interpreted above, the DEM results were confirmed by the quantitative and qualitative experimental results during the molding process, indicating that the DEM simulation could be used to characterize the feed extrusion process and the compression behavior. Therefore, the DEM parameters of feed raw material were reasonable and appropriate.

## Conclusion

1. Based on the JKR contact model in DEM, the contact parameters applied for simulation of the wet-sticky feed raw material samples were selected and calibrated. The Plackett–Burman design experiment and significance analysis were carried out; thus the factors that significantly impact the angle of repose of the feed ingredients were screened as follows: JKR surface energy, MM static friction factor and MM rolling friction, respectively.
2. The three parameters screened out were selected as the influencing factors, and the angle of repose was selected as evaluating indicator; thus the performance optimization experiments were carried out under the quadratic orthogonal rotation design. Taking the experimentally measured angle of repose value of 54.25°as the target value, the significance parameters were optimized, and the optimal combination was obtained : MM rolling friction factor was 0.21, MM static friction factor was 0.51, and JKR surface energy was 0.65. Based on the optimizing combination of influencing factors, the average angle of repose was 53.94° measured by simulated test. The relative error of angle of repose between the simulated and experimental tests was 0.57%.
3. In order to further verify the accuracy and effectiveness of the parameters, the angle of repose and SPP tests were compared under the calibrated parameters. The results showed that the relative error of experimental and simulated tests in angle of repose calibration was 0.57%, and the compression displacement and compression ratio of the experimental and simulated tests in SPP were 1.01% and 0.95%, respectively, which improved the reliability of the simulated results. It has promising prospects in the improvement of feed processing parameters and the optimization of related equipment.

## Data Availability

All data included in this study are available upon request by contact with the corresponding authors.
